# The therapeutic effects of methylphenidate and matrix-methylphenidate on addiction severity, craving, relapse and mental health in the methamphetamine use disorder

**DOI:** 10.1186/s13011-020-00317-y

**Published:** 2020-09-25

**Authors:** Nazanin Aryan, Hamid Reza Banafshe, Vahid Farnia, Jalal Shakeri, Mostafa Alikhani, Habibollah Rahimi, Mojtaba Sehat, Peyman Mamsharifi, Amir Ghaderi, Abdollah Omidi

**Affiliations:** 1 Department of Addiction studies, School of Medical, Kashan University of Medical Sciences, Kashan, Iran; 2 Department of Pharmacology, School of Medicine, Kashan University of Medical Sciences, Kashan, Iran; 3 Substance Abuse Prevention Research Center, Health Institute, Kermanshah University of Medical Sciences, Kermanshah, Iran; 4 Department of Biostatistics and Epidemiology, School of Public Health, Kashan University of Medical Sciences, Kashan, Iran; 5 Department of Community Medicine, Kashan University of Medical Sciences, Kashan, Iran; 6 Department of Psychology, Allameh Tabataba’i University, Tehran, Iran; 7 Clinical Research development unit-Matini/Kargarnejad Hospital, Kashan University of Medical Sciences, Kashan, Iran; 8 Department of clinical psychology, School of Medicine, Kashan University of Medical Science, Kashan, Iran

**Keywords:** Methamphetamine, Methadone, Methylphenidate, Matrix model treatment, Craving, Relapse, Mental health

## Abstract

**Background:**

Little evidence has examined the therapeutic effects of methylphenidate (MPH) and Matrix Model treatment on addiction severity, craving, relapse and mental health in people who use methamphetamine (PWUM). This study was conducted to determine the effects of MPH, Matrix Model treatment, and Matrix-MPH on addiction severity, craving, relapse and mental health in PWUM.

**Methods:**

This clinical trial was conducted among 100 patients with METH users. Participants were randomly divided into four groups who received: 1) 22 sessions of 45-min, twice a week for Matrix Model treatment (*n* = 25); 2) MPH 10 mg/day in the first month, 7.5 mg/day in the second month and 5 mg/day in the third month (*n* = 25); 3) Matrix Model treatment combined with MPH (*n* = 25); 4) control group (*n* = 25) for 12 weeks. Addiction severity, craving, relapse and mental status were evaluated at baseline and end-of-trial.

**Results:**

Matrix Model treatment combined with MPH significantly reduced MA craving (*P* < 0.001) and addiction severity (*P* < 0.001). In addition, Matrix Model treatment combined with MPH resulted in a significant increase of mental health (*P* = 0.001), compared with Matrix Model treatment, MPH, and control group. Also, negative METH urine test significantly increased in the Matrix Model treatment combined with MPH group compared with the other groups (*P* < 0.001).

**Conclusions:**

In conclusion, Matrix Model treatment combined with MPH for 12 weeks had beneficial effects on addiction severity, craving, relapse, and mental health in PWUM, compared with Matrix Model treatment, MPH, and control group.

**Trial registration:**

This study was retrospectively registered in the Iranian website (www.irct.ir) for clinical trials registration (http://www.irct.ir: IRCT20171105037245N1). Registration date: 9 December 2017.

## Background

Methamphetamine (METH) is a widely used illicit drug. In the world, between 162 and 324 million people used an illicit drug (heroin, cannabis, cocaine, and amphetamine type stimulants) at least once in the previous year. METH is a highly addictive psycho-stimulant and a current health concern in Iran [1, 2]. The prevalence of METH dependence is less than 1 % in the general population of Iran [3]. Among of these actions, MMT could be introduced as an effective treatment which enables the enhancement of the quality of life and social functioning [4]. Despite successful implementation of MMT, some barriers and challenges still have remained. Various reports revealed that the prevalence of METH use in Iran has increased among MMT patients [2, 5]. PWUM is associated with several psychiatric impacts such as elevated craving, withdrawal syndrome and relapse [6–8].

Main strategy for managing stimulant disorders is psychosocial interventions (e.g, Matrix Model treatment and the Cognitive Behavioral Therapy) in Iran and in the world. Also, motivational interviewing, cognitive behavioral therapy, family therapy, and contingency management could be employed as treatments for PWUM [9–11]. According to the current evidence, the Matrix Model treatment was provided at a few MMT centers as an approach for treating stimulant disorders, especially cocaine and METH [3, 12, 13]. The Matrix Model helps patients to get necessary and appropriate information to build a healthy life-style support to withdrawal drugs. However, to the best of our knowledge, we did not find any meta-analysis related to the pharmacotherapy or Matrix in PWUM. In a pilot study conducted by Rawson et al. [14], reduction in alcohol and cocaine use and improvement in some psychological symptoms was seen after Matrix Model treatment. In addition, evidence indicated the efficiency of matrix treatment approach in the enhancing self-efficacy and preventing relapse for patients withdrawal METH [15].

Pharmacotherapy has been emerged as a new platform in the treatment of drug use. This therapy can have some benefits. Regardless of various benefits, there are different challenges to utilize pharmacologic components in order to attain the best method of therapy in drug use. From the psychotic disturbance perspective, the impact of MPH on drug use therapy has been confirmed [16]. MPH is a nor-adrenaline and dopamine re-uptake inhibitor with agonist-like activity. As a result, it has the potential to be used as a therapeutically substitute for METH and amphetamine [17]. Tiihonen et al. [18] showed the administration of 54 mg/day MPH compared with aripiprazole to patients with severe amphetamine dependence for 20 weeks led to a significant fewer amphetamine positive urine. Also, evidence studies have demonstrated that both risperidone and MPH can be useful for treatment of PWUM, in order to decrease the drug craving and somatic, psychological, and neurologic problems [19]. However, in another study, MPH administration was associated with no changes in the percentage of positive urines [20]. Matrix model and Methylphenidate have been suggested to act jointly rather than independently. Existing evidence shows that joint Matrix model with Methylphenidate treatment is much more efficient in influencing severity of addiction, craving, relapse and mental health than single Matrix model or MPH treatment.

To our best knowledge, studies demonstrating the effects of MPH, Matrix Model treatment and matrix-MPH on clinical symptoms improvement (e.g., addiction severity, craving, relapse and treatment retention) in PWUM are scarce. Therefore, we hypothesized that joint Matrix model with Methylphenidate treatment is better than single Matrix model or Methylphenidate treatment on addiction severity, craving, relapse and mental health among PWUM.

## Methods

### Trial design and participants

This study was registered in the Iranian website for clinical trials (http://www.irct.ir: IRCT20171105037245N1). This clinical trial was performed on 100 patients with PWUM, 20 to 48 years old, who were referred to the Faraby Hospital Substance Dependency Clinic in Kermanshah, Iran. All participants fulfilled The Declaration of Helsinki requirements and signed an informed consent. This study was approved by the ethics committee of Kashan University of Medical Sciences (IR.Kaums.REC.1396.59).

### Inclusion criteria

Patients were included if they had any of the following criteria: aged 20 to 48 years, METH dependence, as assessed by the substance use section of the Structured Clinical Interview for DSM-IV, Seeking treatment for METH use, at least weekly self-reported METH use during a preceding three month period.

### Exclusion criteria

Patients were excluded if they had any of the following criteria: unwillingness to participate, having severe psychotic disorders in the past 6 months, suicide attempts within the past 12 months, alcohol, sedative physical dependence and cocaine dependence, present or recent use (within 2 weeks) of over-the-counter or prescription drug that would be expected to have major interaction with MPH, major cardiovascular disorder and epilepsy.

### Study design

This study was conducted from November 2017 to February 2018 among volunteers from PWUM in Faraby Hospital Substance Dependency Clinic in Kermanshah, Iran. All patients received MMT program. Methadone was consumed in the form of syrup by patients. The patients were divided into four groups: Matrix Model treatment, MPH, Matrix-MPH and control groups by a trained staff. The first group (25 people) was treated with Matrix Model treatment for 22 sessions of 45 min, twice a week for three months (Table 1) [13, 14, 21]. Also, the second group (25 people) was received MPH 10 mg/day in the first month, 7.5 mg/day in the second month and 5 mg/day in the third month. The third group (25 people) was received Matrix Model treatment combined with MPH. The control group (25 people) had no intervention. MPH tablets were purchased from Novartis Company (Switzerland). The study was explained to each individual before starting and written consent was obtained. The psychiatric and medical information, history of illicit drug use, social status and demographic data of patients were evaluated through a pre-designed structured clinical interview.

**Table 1:** Summary of Matrix Treatment Sessions

Sessions	Content of sessions
**1 session**	Why do we stop taking drugs (scale of change), task assignment
**2 session**	Triggers (stimulating factors), task assignment
**3 session**	External triggers, task assignment
**4 session**	Internal triggers, task assignment
**5 session**	Recovery phase, task assignment
**6 session**	Family distrust, task assignment
**7 session**	Energy reduction, task assignment
**8 session**	Incorrect use of medication, task assignment
**9 session**	Temptation, how to grow up and behave, task assignment
**10 session**	Thoughts and feelings shaping behavioral use, task assignment
**11 session**	Boredom and depression, task assignment
**12 session**	Relapse prevention, task assignment
**13 session**	Work and recovery, task assignment
**14 session**	Shame and guilt, task assignment
**15 session**	Staying busy, task assignment
**16–18 session**	Motivation for recovery, task assignment
**19 session**	Truthfulness, task assignment
**20 session**	Complete innocence, task assignment
**21 session**	Addictive sexual relations
**22 session**	Review of the last session and answering questions

### Randomization

Randomization assignment was done using computer-generated random numbers and was done by a trained staff at the clinic as a blind.

### Outcomes

Craving and addiction severity were considered as the primary outcomes of interest and mental health and relapse were considered as the secondary outcomes.

### Clinical measures

#### Craving

METH craving was assessed with Desire for Drug Questionnaire (DDQ) designed by Franken et al. [22]. DDQ is used for heroin dependents to evaluate heroin craving at the moment. In a study by Franken et al., the total Cronbach’s alpha was reported to be 0.85 for general credit questionnaire. In addition, according to Abharian et al. in 2016, this score was reported to be 0.75 for general credit questionnaire in Persian-speaking users [23].

#### Mental health

##### General health questionnaire (GHQ-12)

GHQ-12 includes 12 items rated on a 4-point Likert scale (0 = Not at all to 4 = Much more than usual). A study on 421 adult outpatients in Germany has reported high internal consistency for GHQ-12 (α = 0.91) [24]. In Iran, psychometric properties of GHQ-12 were assessed by Montazeri et al. The above-mentioned study was conducted among 748 young people, showed that GHQ-12 is well-validated with a Cronbach alpha coefficient of 0.87 [25].

#### Relapse

The patients’ urine tests were examined for the presence of amphetamine, METH, and methadone at the beginning of the study and on every three weeks visit. These tests were performed to assess relapse for patients.

#### Severity of addiction

Leeds Dependence Questionnaire (LDQ) was designed to measure the degree of dependency from mild to severe. It is used to assessment the dependency of various substances. LDQ-10 items are a based on a four-point Likert scale. Cronbach’s alpha was demonstrated to be 0.94 and test-retest reliability was 0.95 [26]. In Iranian population, LDQ was assessed by Habibi et al., and Cronbach’s alpha was 0.90 [27].

### Sample size

In this study, we were used a randomized clinical trial sample size calculation formula where type one (α) and type two errors (β) were 0.05, and 0.20 (power = 80%), respectively. According to our previously published trial [20], we were used s1, s2, μ1 and μ2 of the craving score in the MPH and placebo groups were respectively 32.9, 31.9, 61.7, and 50. Based on the formula, we needed 20 participants in each group. After allowing for 5 dropouts in each group, the final sample size was 25 persons in each group.

### Statistical analysis

The Kolmogorov-Smirnov test was done to determine the normality of data. ANOVA and chi-square test was used to detect differences in general characteristics between the four groups. To determine the effects of MPH and Matrix-MPH on addiction severity, craving and mental health, we used ANCOVA and Bonferoni post hoc pair-wise comparisons. Also, since the patients relapse rate were examined over the study period for five times, logistic regression analysis with GEE approach was run to assess the treatment effects on it, regarding 0 and 1 codes as the positive and negative urine test results, respectively. All statistical analyses were done using the Statistical Package for Social Science version 18 (SPSS Inc., Chicago, Illinois, USA).

## Results

**Fig. 1:**
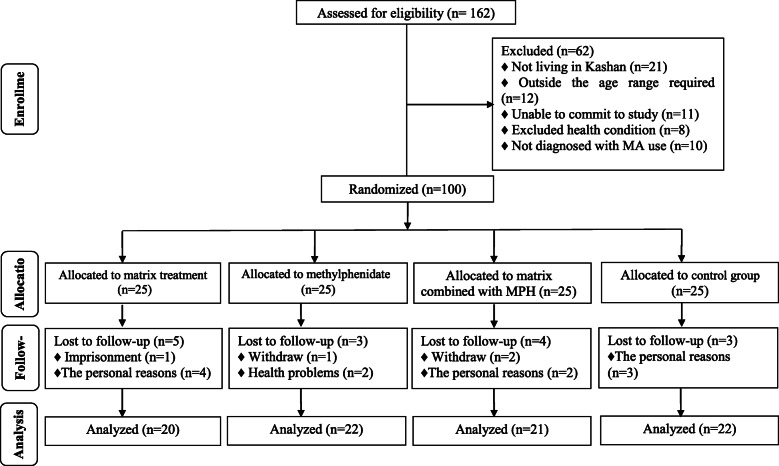
Summary of patient’s flow diagram

Among patients in the Matrix Model treatment group, 5 individuals [imprisonment (*n* = 1) and the personal reasons (*n* = 4)], in the MPH group, 3 individuals [withdraw (*n* = 1) and health problems (*n* = 2)], and in the Matrix Model treatment combined with MPH group, 4 individuals [withdraw (*n* = 2) and the personal reasons (*n* = 2)] were excluded. The exclusions in the control group were 3 patients [the personal reasons (*n* = 3)]. Finally, 85 patients [Matrix Model treatment (*n* = 20), MPH (*n* = 22), matrix combined with MPH (*n* = 21), and control (*n* = 22)] completed the trial (Fig. 1).

In each visit, patients were asked about the side-effects. The grade 1 side-effects were reported following the using MPH in the Matrix Model treatment combined with MPH and MPH group [e.g., loss of appetite, insomnia, abdominal pain, constipation, headache, and dry mouth]. The severity of the side-effects was mild, and which did not lead to excluding any patients from the study (Table 2). Based on the results, MPH was safe by PWUM during MMT programs.

**Table 2:** Frequency of side effects in MPH and Matrix Model treatment combination with MPH

Side Effects	Methylphenidate Group (*n* = 22)	Matrix combination with methylphenidate Group (*n* = 21)
loss of appetite	8 (18.6)	9 (21.4)
Insomnia	6 (14)	4 (9.5)
Abdominal pain	2 (4.7)	1 (2.4)
Constipation	3 (7)	1 (2.4)
Headache	1 (2.3)	2 (4.8)
Dry mouth	6 (14)	8 (19)

All variables are presented as number (%)

Mean age, education status, marital status, job, dose of METH use, duration of METH use, frequency of METH use, duration of MMT, and methadone dose at baseline and end-of-trial were not significantly different between four groups (Table 3).

**Table 3:** General characteristics of study participants^1^

	Matrix group (***n*** = 20)	Methylphenidate group (***n*** = 22)	Matrix combination with methylphenidate group (***n*** = 21)	Control group (***n*** = 22)	P^**2**^
Age (y)	30.7 ± 6.1	31.8 ± 5.6	29.4 ± 2.7	31.0 ± 2.7	0.39
Education status (%)					
Elementary	5 (25)	9 (40.9)	8 (38.1)	7 (31.8)	
Intermediate	8 (40)	4 (18.3)	3 (14.3)	7 (31.8)	
Diploma	5 (25)	6 (27.3)	8 (38.1)	5 (22.7)	0.52†
College	0 (0)	3 (13.6)	1 (4.8)	1 (4.5)	
Bachelor of science (BSc)	2 (10)	0 (0)	1 (4.8)	2 (9.1)	
Marital status (%)					
Permanent marriage	8 (40)	3 (13.6)	6 (28.6)	8 (36.4)	
Single	6 (30)	11 (50)	6 (28.6)	7 (31.8)	0.49††
Widow/Divorced	6 (30)	8 (36.4)	9 (42.9)	7 (31.8)	
Job (%)					
Unemployed	12 (60)	14 (63..6)	7 (33.3)	8 (36.4)	
Employed	2 (10)	1 (4.5)	2 (9.5)	0 (0)	0.09†
Others	6 (30)	7 (31.8)	12 (57.1)	14 (63.6)	
Methadone dose (mL/d)	16.8 ± 4.4	18.6 ± 5.3	18.1 ± 5.7	18.0 ± 3.7	0.67
Duration of MMT (y)	4.7 ± 2.3	4.1 ± 2.0	5.4 ± 2.1	4.1 ± 1.9	0.16
Dose of METH use (g/week)	0.35 ± 0.2	0.43 ± 0.2	0.35 ± 0.2	0.43 ± 0.2	0.55
Frequency of METH use (week)	2.9 ± 1.6	3.7 ± 2.0	3.4 ± 1.8	3.4 ± 1.7	0.53
Duration of METH use (y)	1.8 ± 0.83	2.1 ± 0.94	1.7 ± 0.76	2.3 ± 0.94	0.12

^1^ Data are mean ± SDs

^2^ Obtained from One-Way ANOVA

† Obtained from Fisher’s Exact test

†† Obtained from Pearson Chi-square test

No significant differences were observed among four groups in terms of baseline values of DDQ, LDQ, and GHQ. Matrix-MPH group, compared with Matrix Model treatment, MPH, and control group, led to reduced DDQ score (*P* < 0.001), LDQ score (*P* < 0.001), and GHQ score (*P* = 0.001) (Table 4).

**Table 4:** The effect of Matrix Model treatment, MPH and matrix-methylphenidate on clinical parameters in METH users ^**1**^

Group Variable	Matrix (n = 20)	Methylphenidate (n = 22)	Matrix combination with methylphenidate (*n* = 21)	Control (*n* = 22)	*P*-value^*^	Effect size
DDQ^2^ Baseline	53.4 ± 6.4	53.9 ± 4.8	54.9 ± 4.5	55.9 ± 4.5	< 0.001	0.387
End-of-trial	53.7 ± 5.7^**a**^	51.2 ± 4.0	49.4 ± 5.5	56.4 ± 3.7^**b**^		
*P*-value^**^	0.701	< 0.001	< 0.001	0.291		
LDQ^3^ Baseline	21.2 ± 2.8	20.6 ± 2.6	22.3 ± 2.9	20.8 ± 2.8	< 0.001	0.226
End-of-trial	21.9 ± 2.9^**a**^	19.5 ± 2.3	19.3 ± 1.4	20.2 ± 1.6		
*P*-value^**^	0.410	0.004	< 0.001	0.304		
GHQ^4^ Baseline	31.4 ± 4.9	28.2 ± 6.6	30.9 ± 4.8	31.4 ± 5.3	0.001	0.178
End-of-trial	30.3 ± 4.2	27.6 ± 5.2	27.0 ± 5.0^**c**^	30.6 ± 5.5		
*P*-value^**^	0.096	0.480	< 0.001	0.195		

^1^ Data are mean ± SDs

^2^ DDQ: Desire for Drug Questionnaire

^3^ LDQ: Leeds Dependence Questionnaire

^4^ GHQ: General Health Questionnaire

**a**: Significant difference between Matrix with Methylphenidate and Matrix combination with methylphenidate groups (Bonferroni test)

**b**: Significant difference between Control with Methylphenidate and Matrix combination with methylphenidate groups (Bonferroni test)

**c**: Significant difference between Matrix combination with methylphenidate with other groups (Bonferroni test)

* ANCOVA test/ ** Paired t-test

In four groups, all METH urine tests were positive in the beginning of the trial. Over the next few weeks, METH Positive Urine test (MPUT) were gradually reduced in four groups, but the reduction was greater in Matrix-MPH group compared with other groups. Twenty percent (4/20) in Matrix Model treatment group, 40.9% (9/22) in MPH group, 61.9% (13/21) in Matrix-MPH group, and 5% (1/22) in the control group had negative METH urine tests in the last week. In addition, our study revealed that there was a significant effect in METH positive urine tests among four groups (P < 0.001). GEE logistic regression model revealed Matrix Model treatment combined with MPH had an effective treatment on relapse rate with odds ratio 7.63 (CI; 2.82–20.67) compared with other groups (Table 5).

**Table 5:** The effect of Matrix Model treatment and MPH on METH Positive Urine test (MPUT) ^1^

Variable	Group	Baseline	Week 3	Week 6	Week 9	Week 12	*P*-value^*^	OR (95% CI)
MPUT	**Matrix (*n*** **= 20)**	20 (100)	19 (95)	18 (90)	16 (80)	16 (80)^**a**^	< 0.001	2.59 (0.87–7.75)
	**Methylphenidate (*n*** **= 22)**	22 (100)	20 (90.9)	18 (81.8)	16 (72.7)	13 (59.1)		4.95 (1.79–13.68)
	**Matrix combination with methylphenidate (*n*** **= 21)**	21 (100)	19 (90.5)	17 (81)	12 (57.1)	8 (38.1)		7.63 (2.82–20.67)
	**Control (*n*** **= 22)**	22 (100)	21 (95.5)	21 (95.5)	20 (90.9)	21 (95.5)^**b**^		Reference

^1^ All variables are presented as number (%)

* GEE analysis (generalized estimating equations)

**a**: Significant difference between Matrix and Matrix combination with methylphenidate groups

**b**: Significant difference between Control with Methylphenidate and Matrix combination with methylphenidate groups

## Discussion

The current study compared the efficacy of two therapies MPH and Matrix-MPH on reducing METH craving, addiction severity, relapse, and mental health in PWUM. Results showed that Matrix-MPH was effective in reducing METH craving, addiction severity, relapse, and improvement of mental health symptoms. In the current study, the effect size of LDQ and GHQ was nearly 0.2 means that the score of average person in the co-treatment group was nearly 0.2 standard deviations above the average person in other groups, and hence exceeds the scores of 58% of other groups. In addition, the effect size of DDQ was nearly 0.38 means that the score of the average person in the co-treatment group was 0.38 standard deviations above the average person in other groups, and hence exceeds the scores of 68% of other groups. It must be kept in mind that in the current study, METH use parameters at baseline demonstrated that individuals were using METH for a maximum of 2.3 years. Long-term PWUM may lead to abnormal findings in CNS, renal, skin, and gastrointestinal. Therefore, METH heavy users for a longer time may require longer treatment duration. Previous study showed that mental and clinical disturbances were present in PWUM under MMT. Based on these findings, Matrix Model treatment combined with MPH may be an appropriate adjunct therapy for PWUM. To our best knowledge, this clinical trial for the first time evaluated the effects of Matrix Model treatment and MPH on craving, addiction severity, relapse, and mental health in PWUM.

### Effects on mental health

Addiction is a mental disturbance that influences all aspects of life, family, individual health, and society [28]. PWUM under methadone therapy program are susceptible to several mental disturbances (e.g., depression, and anxiety) [29, 30]. We found that Matrix Model treatment, Matrix Model treatment combined with MPH, and MPH to PWUM in methadone therapy program for 12 weeks improved general health indexes. Few studies have shown the beneficial effects of Matrix Model treatment and MPH on psychological parameters in subjects without METH users under methadone therapy program, though the results are controversial. The Matrix Model treatment applies to mental health systems of care [31–33]. Masaeli et al. [34], shown that Matrix Model treatment intervention is effective in improving anxiety, depression and quality of life in PWUM, as well as their caregivers. Also, previous evidences demonstrated the beneficial effects of cognitive methods on posttraumatic stress disorder (PTSD) symptoms, and depression signs in non-amphetamine users [35, 36]. In two randomized controlled trials, MPH administration among subjects with terminal disease had beneficial effects on fatigue symptom, and depression rating scales [37, 38]. In addition, MPH intake ameliorated clinical symptoms (e.g. apathy, depression, and activities of daily living scales) in elderly patients with dementia of Alzheimer type [39]. However, MPH administration showed no changes in anxiety state by children suffering from attention deficit hyperactive disorder [40]. In explaining the effectiveness of Matrix Model treatment interventions on complications related to anxiety and mood, different factors involved in treatment process such as increasing social support, problem-solving skill, life style changes, behavior change, education about dependencies, training in relapse prevention, and family involvement which may decrease the patients’ mood problems [11, 41]. MPH may improve rating scales in mental health through regulating neurotransmitters in the brain such as dopamine, serotonin and norepinephrine [42].

### Effects on craving, addiction severity, and relapse

Craving, withdrawal syndrome, physical, and psychological harms have been linked to amphetamine/methamphetamine use, especially with an enhancing frequency of use [6, 43, 44]. Our study supported that Matrix Model treatment combined with MPH for 12 weeks by PWUM under methadone therapy significantly reduced craving, relapse, and addiction severity. Some behavioral approaches including contingency management, cognitive behavior therapy, and the Matrix Model treatment might have effect on the treatment of PWUM. The Matrix Model treatment is an individualized outpatient regimen that has been used successfully to treat patients who use stimulants which is based on cognitive principles [45, 46]. The efficacy of Matrix Model treatment may be useful for amphetamine and cocaine dependence, and providing treatments for a longer time and developing efficacious relapse prevention strategies [13, 47, 48]. Some randomized placebo-controlled clinical trials showed the positive effect of MPH and Matrix Model treatment on craving, addiction severity, and relapse. It has been revealed in a study by Dolan that treatments should target self-efficacy in cocaine addict patients [49], and Matrix Model treatment helps improving self-efficacy in adolescents with substance use. In addition, Matrix Model treatment for 16 weeks by patients with cocaine-dependent had beneficial effects on better abstinence outcomes [50]. However, Matrix Model treatment showed no changes in any of the subscales of addiction severity between male and female METH users [21]. Matrix Model treatment acts as a comprehensive therapy that contains all necessary skills that both the family and the patient must learn to challenge life problems [13]. In Iran, about half of the centers offered METH psychological and pharmacological treatment services, although 89% of the therapeutic options focused on Matrix Model treatment [47]. Until now, no pharmacotherapy was associated with sufficient results and consistent evidence of effectiveness to support its use in routine treatment of PWUM [51]. Previously, it was reported that MPH has beneficial effects in subjects with ADHD. In addition, MPH may improve treatment retention resulting in reduced drug use that suggested higher doses may be optimal for some groups of amphetamine dependent [51–53]. Four studies used the control-released MPH (using 18, 36, 54 mg/day at the first, the second, and 7–17 weeks respectively) than placebo [18, 20, 54, 55]. Two studies indicated that MPH compared to placebo can decrease the number of amphetamine-positive urine samples [18, 54]. In third trial the self-reported days of PWUM [55], but not in a fourth study [20]. In has been shown that the MPH at 10 mg/day is able to decrease the craving symptoms less than risperidone [19]. The primary pharmacologic effect of MPH is to enhance nor-epinephrine activity and dopamine, which impacts reward system function. MPH actions such as nor-epinephrine and dopamine transporter inhibition, redistribution of the VMAT-2, and agonist activity at the serotonin type 1A receptor [56, 57]. Future evidence should be developed focusing on efficacy and long-term safety in PWUM under MMT program.

### Limitations

The present study had some limitations. Duration of this study was short. Long-term duration may lead to better effects. Also, we did not evaluate the effects of Matrix Model treatment and MPH on metabolic profiles and cognitive function. In addition, we could not evaluate the pain in our methadone therapy program. Another limitation was that no female subjects were in the project, as cultural considerations tend to prevent women from referring to MMT clinics in Iran.

## Conclusions

In summary, the combination of the Matrix Model treatment with MPH in PWUM under a MMT program had a beneficial effect on addiction severity, craving, relapse, and mental health parameters. Further studies are needed to show the relative impact of Matrix Model treatment and MPH on PWUM under MMT program.

## Data Availability

The primary data for this study is available from the authors on direct request.

## Abbreviations

DDQ: Desire for Drug Questionnaire
GHQ: General Health Questionnaire
LDQ: Leeds Dependence Questionnaire
MPUT: METH Positive Urine test
